# Advances in understanding the mechanisms of the human papillomavirus oncoproteins

**DOI:** 10.1042/BST20253041

**Published:** 2025-05-16

**Authors:** Denise Ijeoma Obanya, Louisa M. Wootton, Ethan L. Morgan

**Affiliations:** School of Life Sciences, University of Sussex, Brighton, U.K

**Keywords:** cellular plasticity, E6, E7, epigenetics, genomic instability, HPV, ncRNA

## Abstract

High-risk human papillomaviruses (HPVs) are responsible for almost all cervical cancer cases and a growing number of oropharyngeal and anogenital cancers. The primary HPV oncoproteins, E6 and E7, act together to manipulate multiple cellular pathways that can ultimately result in malignant transformation. This includes the deregulation of several signalling pathways that regulate cell proliferation, cell cycle progression and cell survival. Although multiple functions of HPV E6 and E7 in driving oncogenesis are well known, recent studies have uncovered novel oncogenic functions of the HPV oncoproteins, including the manipulation of emerging mechanisms of cancer development, such as epigenetic modifications, cellular plasticity and genomic instability. This review explores current advances in understanding how the HPV oncoproteins interact with these cellular processes, highlighting potential therapeutic targets in HPV-associated cancers.

## Introduction

Around 15% of cancers are caused by pathogens, with around a third of these caused by human papillomavirus (HPV) infection [1]. HPVs are a diverse group of DNA viruses that infect host keratinocytes in the stratified epithelia [2,3]. The majority of HPVs have a common genome organisation and express a similar pattern of viral genes [4–6]. HPVs can broadly be classified as high-risk (HR) or low-risk based on their association with the development of malignancies [7]. Whilst the association between HR-HPV infection and cancer development is well established, only 10–20% of infections persist and lead to malignancy [8]. To date, around 15 HR-HPV types have been identified, with HPV16 and HPV18 being the most common, accounting for about 70% of cervical cancers [5]. Additionally, HR-HPV infection is associated with four other ano-genital cancers (anal, penile, vaginal and vulvar) and oropharyngeal cancers [7]. The primary drivers of HPV-mediated oncogenesis are the major oncoproteins E6 and E7, with the minor oncoprotein E5 playing a smaller, supporting role [9]. These proteins deregulate multiple signalling pathways and cellular processes, providing an environment conducive to allow viral replication and persistence. HPV infection drives cell cycle progression, proliferation and survival through the function of these proteins, ultimately promoting malignant progression [5]. The most well-studied mechanisms are the degradation of the tumour suppressor retinoblastoma protein (pRb) by E7, causing aberrant cell cycle entry via deregulation of the G1/S checkpoint [10,11], and the E6-mediated degradation of p53, promoting continuous proliferation and survival [12,13]. The oncogenic mechanisms of HPV E5 are less well studied, but it plays a role in promoting proliferation, primarily via epidermal growth factor receptor (EGFR) signalling [14,15]. The mechanistic understanding of how the HPV oncoproteins function to promote oncogenic development has been greatly expanded over the past 40 years. However, novel functions continue to be uncovered, particularly as new oncogenic mechanisms in general are discovered as technical advances are made. Considering this, here we highlight recently discovered pro-oncogenic functions of HPV E6 and E7, particularly in the areas of epigenetics, genomic instability and cellular plasticity, which have become increasingly recognised as important drivers of malignant development in multiple cancers [16–18].

## Epigenetic modifications

Epigenetic modifications can result in changes in gene expression that occur independently of changes to the DNA sequence of the gene [19]. These heritable changes can have a significant impact on the temporal and spatial control of gene expression. Therefore, dysregulation of epigenetic mechanisms can result in the development of diverse diseases, including cancer [20]. Several epigenetic mechanisms have been identified to date that can be categorised based on one of the five principal mechanisms: DNA methylation/modification, histone modification, RNA methylation/modification, chromatin remodelling and regulation by non-coding RNAs (ncRNA). Multiple interactions of the HPV oncoproteins with the epigenetic machinery have been identified, and extensive epigenetic alterations have been observed in HPV-associated cancers [16,21,22].

### DNA methylation

DNA methylation, the addition of a methyl group to the fifth carbon of cytosine residues (5-methylcytosine, 5mC), is catalysed by DNA methyltransferases (DNMTs) and regulated by methyl-binding domain-containing proteins and demethylases [19]. In normal cells, DNA methylation regulates gene expression, including the organisation of active and inactive chromatin, tissue-specific gene expression and genomic imprinting. In contrast, both hypomethylation and hypermethylation can play a crucial role in cancer development by regulating the expression of oncogenes or tumour suppressors. The expression of DNMT1, a maintenance methyltransferase, is often increased in cancer, demonstrating the importance of DNA methylation in malignant development [23]. Early studies demonstrated that both HPV E6 and E7 can regulate DNMT1 expression and that HPV E7 can interact with DNMT1, promoting its activity [22,24,25]. Furthermore, HPV E7 can induce the expression of DNMT3a and 3b, *de novo* DNMTs [26]. This can result in the regulation of multiple genes that contribute to oncogenesis, including *CDH1* [26,27], *CCNA1* [28], *RASSF1*A [29], *DAPK1, MGMT* and *RARB* [30]. DNA methylation can also regulate the HPV upstream regulatory region (URR); hypermethylation of the E2 binding site 1 (E2BS1) results in the activation of the URR, promoting E6 and E7 expression [31]. Futhermore, DNA methylation has also been suggested as a novel biomarker in HPV screening [32]. The cell adhesion molecule 1 (*CADM1*) gene has been identified as a potential biomarker in multiple HPV-associated cancers [33–35]. *CADM1* is frequently silenced by promoter hypermethylation during cervical disease progression. A recent study demonstrated that HPV18 infection mediates the repression of *CADM1* via topological rearrangement of the *CADM1* locus; loss of CCCTC-binding factor (CTCF) binding at the *CADM1* locus precedes hypermethylation of the *CADM1* transcriptional promoter, demonstrating a novel mechanism of HPV-induced host cell transcriptional reprogramming that may promote oncogenesis [36]. As an extra level of epigenetic regulation, 5mC can be oxidised to 5-hydroxymethylcytosine, 5hmC [37]. These epigenetic marks can be differentially regulated by different ‘reader’ proteins. Methyl-CpG-binding domain (MBD) proteins 2 and 3 regulate gene expression by binding to 5mC or 5hmC, respectively [38]. The 5hmC loss has previously been associated with the progression of cervical intraepithelial neoplasia, suggesting it may play a role in cervical cancer development [39]. Mechanistically, HPV E7 down-regulates MBD2 expression and promotes ten-eleven translocation enzymes 1 (TET1) up-regulation, a key gene involved in the oxidation of 5mC to 5hmC [40,41]. This was shown to increase global hydroxymethylation in E7-expressing cells, suggesting a novel mechanism of E7-induced carcinogenesis.

### Histone modification and chromatin remodelling

In addition to DNA methylation, gene expression is also affected by histone modifications, such as the post-translational modifications of histone tails (including acetylation, methylation, phosphorylation, sumoylation and ubiquitination). These modifications can lead to transcriptional activation or repression, depending on the residues modified [42]. These processes can be controlled by several chromatin remodelling complexes, such as the Switch/Sucrose Non-Fermentable (SWI/SNF), the INO80 and the nucleosome remodelling and deacetylation (NuRD) complexes [43]. These multi-subunit complexes contain enzymatic components that function as histone-modifying enzymes, as well as proteins that bind to specific proteins or modifications. For example, the NuRD complex contains histone deacetylase 1 or 2 (HDAC1/2), RbAp46, a histone binding protein, and CDH3 or 4 (also called Mi2α or Mi2β), which are chromodomain helicase DNA-binding proteins [44]. HPV E6 and E7 manipulate several proteins involved in histone acetylation and methylation, including the histone acetyltransferases p300 and Tip60 [45–48]. HPV E7 can also inhibit the function of HDACs by displacing their binding to transcription factors such as HIF1α [49] and E2F2 [50]. Polycomb repressive complex 2 methyltransferase enhancer of zeste homologue 2 (EZH2), a lysine 27-specific histone methylase, is transcriptionally up-regulated by HPV E7 in a pRb/E2F-dependent manner, driving HPV-cancer cell growth [51]. Furthermore, the E7-dependent induction of SUV39H1, a K9-specific methylase, promotes H3K9me2 and H3K9me3 marks, resulting in the repression of the innate immune genes *DDX58* (RIG-I), *CGAS* and *STING1*, providing a novel target to promote the effect of immunotherapies in HPV+ cervical cancer [52]. Conversely, E7 drives a decrease in H3K27Me3 via induction of KDM6A and KDM6B, K27-specific histone demethylases, resulting in host cell transcriptional reprogramming [53]. More recently, HPV+cervical cancer cells have been shown to be dependent on KDM6A expression to survive HPV E7-induced replication stress [54]. Another histone demethylase, KDM2B, was shown to be induced by HPV E6/E7 via cMYC-mediated repression of miR-146a, which subsequently down-regulated KDM2B expression and promoted HPV+cervical cancer cell proliferation [55]. Additionally, KDM5C is degraded by HPV E6, resulting in the up-regulation of the oncogenes *EGFR* and *MET* via deregulated methylation at their super-enhancers, thereby promoting tumourigenesis [56]. HPV can also induce changes in the composition of chromatin remodelling complexes. In E7 expressing cells, the binding of E7 to the stem cell associated transcription factor Octamer-binding transcription factor 4 (OCT4) can result in the formation of a NuRD complex variant containing MBD3 instead of MBD2, affecting downstream transcriptional output [40]. Another study demonstrated that HPV E6/E7 expression can alter the global chromatin deposition binding of the NuRD complex [57], further demonstrating that HPV can drive transcriptional changes via modification of the NuRD complex, both physically and via its chromatin binding ability.

### RNA methylation

N^6^-methyladenosine (m6A) is the most prevalent and abundant RNA modification in eukaryotes, constituting around 60% of RNA methylation [58]. m6A modification is a dynamic and reversible epigenetic mark that is biologically regulated by methyltransferases (‘writers’) and demethylases (‘erasers’), with RNA-binding proteins (‘readers’) binding to the m6A mark and playing an essential role in regulating the expressions and functions of RNA [58]. Due to advances in experimental techniques to identify RNA methylation, its dysregulation in multiple cancers, including cervical cancer, has become apparent in recent years [59]. Methyltransferase-like 3 (METTL3), alongside METTL14 and WT1 associated protein, is a critical ‘writer’ of m6A methylation [59]. m6A modifications are found at higher levels in cervical cancer than in normal cervical tissue [60]. Furthermore, HPV *E7* mRNA transcripts are extensively modified by m6A [61]. These m6A transcripts are bound by the ‘reader’ protein insulin-like growth factor 2 mRNA-binding protein 1 (IGF2BP1), which stabilises the *E7* transcript, promoting oncogenesis [61]. Additionally, HPV E6/E7 expression drives IGF2BP1 expression, stabilising cMyc and promoting HPV-induced tumourigenesis [62]. Furthermore, METTL3 promotes glycolysis in cervical cancer via the stabilisation of hexokinase 2 in a YT521-B homology (YTH) N6-methyladenosine RNA-binding protein F1 (YTHDF1)-dependent manner [60]. METTL3 also promotes the expression of pyruvate dehydrogenase kinase 4 and thioredoxin domain-containing protein 5 in cervical cancer, with METTL3 expression correlating with HPV infection status [63,64]. Finally, a recent study demonstrated that METTL3-mediated m6A modification of the circular RNA circSTX6, which promotes JAK2/STAT3 signalling and HPV-induced transformation, has been previously shown [65–67]. AlkB homologue 5 (ALKBH5), an m6A demethylase ‘eraser’ protein, removes m6A modifications in a one-step catalytic process. HPV E6/E7 induces global m6A modifications and increased ALKBH5 expression through E2F1- and DDX3-mediated transcription, promoting p21-activated kinase 5 expression and cervical cancer progression [68]. However, another study demonstrated that while global m6A modifications are increased in cervical cancer, ALKBH5 expression was decreased, driving Sirtuin 1/acetyl-CoA carboxylase 1 signalling and promoting fatty acid metabolism to regulate tumourigenesis [69]. Thus, further research on the interplay between the writers, readers and erasers of m6A modifications in HPV-associated cancers is warranted [59].

### Non-coding RNAs

ncRNAs, including microRNAs (miRNAs), long non-coding RNAs (lncRNAs) and circular RNAs (circRNAs), have emerged as key regulators of gene expression, affecting oncogenic processes, such as cell proliferation, apoptosis and metastasis [70]. Both HPV E6 and E7 can modulate the miRNA network as a mechanism to control host gene expression [71,72].

### miRNAs

Several miRNAs have been shown to play important roles in HPV-induced cellular transformation, including miR-18a [73,74], miR-21 [75,76], miR-203 [77,78] and miR-375 [79]. Many of these can regulate the expression of E6 and E7 themselves. miR-375 directly targets the *E6* and *E7* transcripts, decreasing their expression [79]. Furthermore, miR-139 has also been shown to down-regulate E6 and E7 expression; however, a direct interaction with the viral transcripts has not been shown [80]. Both miR-139 and miR-375 are down-regulated in HPV+cancers, suggesting that they may play a key role in these cancers [80,81]. Recently, a study performed a screen to identify miRNAs that stabilise p53 in HPV+cervical cancer cells. This identified several miRNAs that also regulated HPV E6 and E7 expression, including the previously identified miR-375 [82]. This study identified miR-519b and miR-1287 as potential novel regulators of HPV E6/E7, but further studies are required to identify the mechanisms involved [82].

### lncRNAs

LncRNAs are ncRNA transcripts with a length greater than 200 nucleotides that contribute to many biological processes by forming complexes with RNAs, DNAs and proteins [83]. The HPV E7-induced lncRNA HOX transcript antisense RNA has been shown to promote proliferation and survival by functioning as an miRNA sponge against miRNAs, such as miR-143 and miR-214, which target *BCL2* and *CTNNB1,* respectively [84–86]. MALAT1, induced by HPV E6 and E7, also promotes proliferation and survival of HPV+cervical cancer via acting as an miRNA sponge [84,87]. Several novel lncRNAs involved in HPV-associated carcinogenesis have been identified in recent years. Thymopoietin pseudogene 2 (TMPOP2), also called lncRNA-EBIC (EZH2-binding lncRNA), is highly expressed in cervical cancer. TMPOP2 is up-regulated by E6/E7 expression and sequesters miR-139 and miR-375, preventing *E6* and *E7* mRNA repression, ultimately promoting HPV-induced proliferation [88]. The lncRNA damage-induced non-coding (DINO) is up-regulated by p53 in response to DNA damage [89]. HPV E7 induces DINO expression in a p53- and KDM6A-dependent manner, and DINO expression stabilises p53 in E7-expressing cells [90,91]. However, in HPV+cervical cancer cells, E6-mediated p53 degradation prevents DINO up-regulation, and exogenous DINO expression causes sensitisation of cervical cancer cells to chemotherapies via induction of p53 and the DNA damage response. HPV can also induce lnc-FANCI-2 expression, which is primarily driven by HPV E7 in a miR-29a/YY1-dependent manner and is up-regulated in cervical cancer [92]. A subsequent study demonstrated that lnc-FNACI-2 is required for the regulation of the Rat Sarcoma Virus (RAS) signalling pathway, suggesting lnc-FANCI-2 has a complex role in the development of cervical cancer [93].

### CircRNAs

CircRNAs are covalently closed RNA molecules that originate from non-sequential back-splicing of exons and/or introns of precursor mRNAs (pre-mRNAs) that primarily function as miRNA sponges, preventing them from exerting their regulatory functions [94]. In cervical cancer, multiple circRNAs are up-regulated. One of these circRNAs, hsa_circ_0018289, promotes cervical cancer cell proliferation *in vitro* and *in vivo*, potentially via sponging miR-497, which functions as a tumour suppressor in cervical cancer [95]. Additionally, circNRIP1 has been shown to promote tumourigenesis in cervical cancer by sponging miR-629-3p, which directly targets protein tyrosine phosphatase 4A1 (*PTP4A1*) mRNA and regulates the MEK/ERK signalling [96]. Recently, it has been demonstrated that HPV16 (and potentially HPV18) expresses circRNAs such as circE7 [97]. CircE7 is highly m6A modified and is translated to produce E7 protein; the disruption of circE7 inhibits E7 expression and cervical cancer cell growth [97]. Further studies demonstrated that circE7 suppresses T cell activity in head and neck squamous cell carcinoma via the down-regulation of *LGALS9,* encoding for galectin-9, which plays a key role in promoting the anti-tumour effect of T cells [98]. However, the biological activity of circE7 has been questioned as another group determined that the effects observed may be attributed to off-target effects on E6*I RNA expression by the circE7 siRNAs used [99]. Therefore, further studies on the role of circE7 are critically important.

## Genomic instability

The DNA damage response (DDR) protects the host genome against mutations caused by DNA damage. Defects or deregulation of the DDR can lead to the accumulation of mutations, causing genetic instability that can drive cancer development. HPV activates the DDR to facilitate efficient viral replication, and this can promote genomic instability [100–102]. HPV E6 and E7 activate multiple DNA pathways that promote viral replication and have implications in HPV-induced carcinogenesis, including Ataxia-telangiectasia mutated (ATM), Chk2 [103] and Ataxia-telangiectasia and Rad3-related protein (ATR) [104]. In addition, viral integration into the host genome directly triggers genomic instability, which may also be due to uncontrolled E6 and E7 expression [105]. The disruption of normal DDR signalling has been observed in HPV E6 and E7 expressing cells, and this likely contributes to increased genomic instability. Both E6 and E7 expressing cells have been shown to have defects in the homologous recombination, nonhomologous end-joining (NHEJ), Fanconi anaemia and translesional synthesis pathways, driving genomic instability in HPV-containing cells [106–111]. However, a recent study comparing the major repair pathways (homologus recombination, NHEJ and microhomology-mediated end-joining [MMEJ]) demonstrated that HPV E7 promotes homologus recombination and MMEJ, while inhibiting NHEJ, potentially in an Rb-dependent manner [112]. Thus, the E7-induced pathway shifts from NHEJ to error-prone MMEJ could induce increased genomic instability in HPV-associated cancers [113]. HPV E7 can bind to and co-opt the E3 ubiquitin ligase RNF168, which is required for proper DNA repair following DNA double-strand breaks (DSBs). RNF168 is expressed at high levels in HPV+cancers, and E7 hinders the function of RNF168 at DSB, promoting genomic instability [114]. HPV E6 and E7 additionally induce mitotic defects, leading to aneuploidy and chromosomal instability. Both proteins induce centrosome amplifications and chromosomal alterations, resulting in numerical and structural chromosomal instability [115–117]. Cosper et al. recently showed that HPV16 E6 causes the specific degradation of centromere protein E, which results in misaligned chromosomes at the spindle pole and a failure in chromosome congression, identifying a novel HPV-induced mechanism of chromosomal instability [118]. Aberrant firing of replication origins results in replication stress, a frequent hallmark of cancer cells that is highly up-regulated in HPV+cancers [119]. Replication stress induces DNA damage, the activation of the ATM and ATR signalling, and subsequent genomic instability. HPV E7 is known to induce replication stress due to aberrant E2F activity as a consequence of pRB inactivation and through other mechanisms [120]. As a consequence of replication stress, HPV E6 and E7 up-regulate the expression of multiple genes to allow continued survival in conditions of high replication stress [119]. HPV hijacks the KDM6A/p21 pathway to tolerate persistent E7-induced replication stress due to p21-mediated Proliferating Cell Nuclear Antigen (PCNA) inhibition [54].

## Cellular plasticity

The complexity of cancer is due to the presence of intratumour heterogeneity, variability within a tumour that arises from diverse populations of cancer cells [121]. Cellular plasticity is key to the development of intratumour heterogeneity, the ability of cancer cells to undergo significant molecular and phenotypic alterations. This allows tumours to adapt to a changing environment, promoting cancer progression, metastasis and therapeutic resistance [122,123]. A classic example of cellular plasticity is epithelial-to-mesenchymal transition (EMT), a process in which epithelial cells gain characteristics of mesenchymal cells. EMT occurs during normal embryonic development and tissue regeneration [124]; however, EMT also occurs during cancer progression in a highly deregulated manner [124]. This allows cancer cells to become highly adaptable to the tumour microenvironment and to become more invasive with a high metastatic potential. Multiple signalling pathways regulate EMT, including the Transforming growth factorβ (TGFβ), Epidermal Growth Factor Receptor (EGFR), Neurogenic locus notch homolog protein (NOTCH), Janus Kinase/Signal transducer and activator of transcription (JAK/STAT), Hippo and WNT signalling pathways [125]. These pathways promote growth and developmental pathways in normal tissues, but can also drive multiple oncogenic phenotypes, such as hyper-proliferation and survival [126,127]. These pathways can also induce expression of the so-called EMT transcription factors (EMT-TF), including SNAIL, Twist-related protein 1 (TWIST) and Zinc finger E-box-binding homeobox 1 (ZEB1), which repress key epithelial markers like E-cadherin, leading to loss of cell–cell adhesion and enhanced migratory potential [121]. Both E6 and E7 can induce EMT via various mechanisms. Phosphoinositide 3-kinase/AKT (PI3K/AKT), STAT3, WNT, Mitogen-activated protein kinase (MAPK) and Transforming growth factor/Mothers against decapentaplegic homolog (TGF/SMAD) signalling all contribute to HPV-induced migration and EMT [128–133]. The Hippo pathway has been shown to play a critical role in regulating HPV-driven tumourigenesis, including invasive phenotypes and EMT [134–136]. EGFR-induced Yes-associated protein 1 (YAP1) activation drives cervical cancer proliferation and migration [134], whereas Transcription Adaptor putative Zinc finger (TAZ) demonstrated HPV-type specific up-regulation of the poorly understood gene Tumour overexpressed gene (TOG) Array Regulator Of Axonemal Microtubules 2 (TOGARAM2) [135]. Additionally, HPV E6/E7 promotes EMT via ΔNp63γ-mediated transcription of the EMT-TF SLUG [137]. p63 isoforms are often amplified and/or overexpressed in HPV+squamous cell carcinomas, suggesting they play a key role in EMT and the invasive phenotypes observed in these cancers.

Cancer stem cells (CSCs), a subpopulation of tumour cells with self-renewing and differentiating capabilities, contribute to tumour initiation, metastasis and therapy resistance [138]. In HPV-associated cancers, CSCs are key drivers of progression [139]. E6 and E7 facilitate CSC formation and maintenance by disrupting tumour suppressor pathways, particularly p53 and pRb, which leads to increased expression of the stem cell-associated transcription factors, including Nanog, SRY-box 2 (SOX2) and OCT3/4 [125,140–142]. Several other pathways have also been shown to reactivate OCT4 expression in cervical cancer. HPV E6 and E7 promote OCT4 expression, and E7 down-regulates MBD2 expression and promotes TET1 expression, deregulating methylation at the *OCT4* promoter [40,143]. E7 further interacts with OCT4 to regulate transcriptional activity, reinforcing stem-like properties and promoting cellular plasticity in HPV+cervical cancer [143]. E6 also contributes to OCT4 expression by repressing p53-dependent transcription via the co-repressor Nuclear receptor co-repressor (NCOR) [144]. Several signalling pathways contribute to ‘stemness’ in HPV+cancers. HPV E6 contributes to up-regulated Hairy and enhancer of split 1 (HES1), a NOTCH target gene that promotes stemness and self-renewal [145]. The expression of YAP, a key transcription factor in the Hippo pathway, in E6/E7-containing oral epithelial cells was shown to induce the rapid expansion of a highly proliferative stem cell-like population, promoting tumour development [146]. Furthermore, the disruption of the interaction between HPV E7 and Tyrosine-protein phosphatase non-receptor type 14 (PTPN14), an endogenous YAP inhibitor that E7 targets for degradation, inhibits cell proliferation and stemness-like properties of HPV+cervical cancer cells [147]. Together, these studies demonstrate the important role of YAP in promoting tumourigenesis, stemness and cellular plasticity in HPV+cancer cells**.**

## Summary

**Figure 1:**
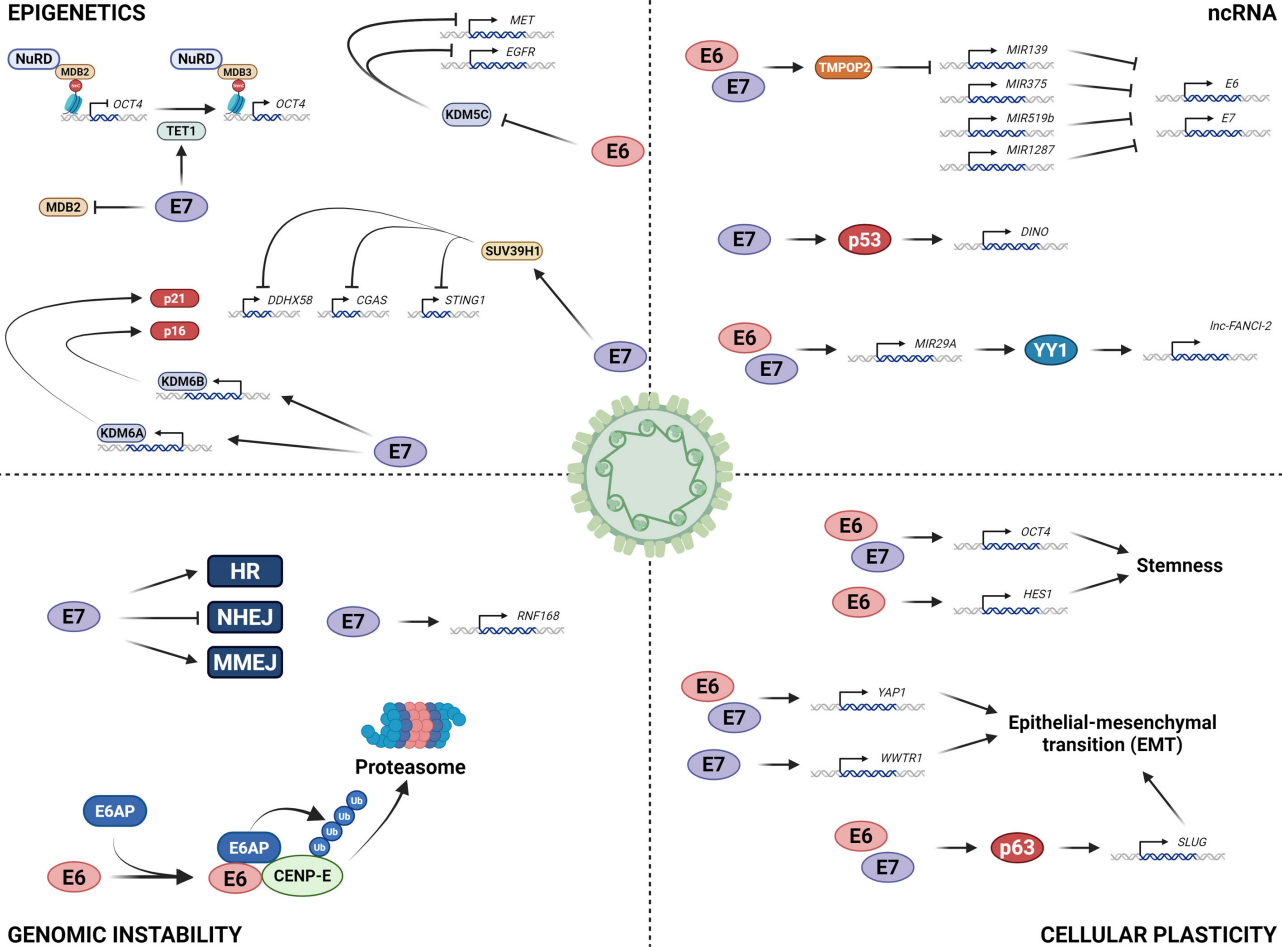
Novel interactions of the human papillomaviruses (HPV) oncoproteins with cellular processes involved in HPV-induced oncogenesis.

HPV E6 and E7 have evolved to hijack many host signalling pathways in order to generate a cellular environment conducive for virus replication. The requirement of these proteins to induce cell cycle progression, proliferation and survival results in the consequential acquisition of mutations in cellular genes, particularly in the context of dysregulated oncoprotein expression, which can facilitate transformation. Although much progress has been made in understanding the host pathways modulated by HPV, novel mechanisms of oncogenesis continue to be elucidated (Figure 1). Many of the mechanisms discussed in this mini-review are potential therapeutic targets for HPV+cancers. Small molecule inhibitors targeting epigenetic regulators, such as DNMTs, HDACs and EZH2, are in various stages of clinical trials for a number of cancers, suggesting that trials to test their efficacy in HPV+cancers are warranted [148]. Moreover, RNA-based therapeutics have become an increasingly viable treatment option in oncology in the last 5 years. Antisense oligonucleotides, RNA interference, aptamers and CRISPR/Cas9-based gene editing offer additional mechanisms to target these pathways as potential cancer therapies [149,150].

Cellular plasticity and the induction of ‘stemness’ can ultimately lead to therapeutic resistance and tumour persistence [151]. As such, targeting the signalling pathways leading to the development of these phenotypes could sensitise cancers to current therapeutics such as chemotherapy and radiation treatment [151]. In HPV+cancers, the induction of cellular plasticity and ‘stemness’ can promote therapeutic resistance [136]. Therefore, targeting pathways that contribute to this phenotype, such as the NOTCH, Wnt and Hippo pathways, may sensitise HPV+cancers to current therapies.

**Perspectives**

- Human papillomavirus (HPV) is an important aetiological cause of cancer, and the function of the HPV oncoproteins E6 and E7 is essential for its ability to promote malignancy.
- Despite the fact that the role of E6/E7 in cancer development has been extensively studied, novel functions continue to be identified. Uncovering how HPV E6/E7 interacts with these emerging biological mechanisms may lead to the identification of novel treatments for HPV+cancers.
- Future studies should focus on understanding these novel mechanisms of E6/E7 function and identifying how to translate these findings into the clinic.

## Abbreviations

ALKBH5: AlkB homologue 5
CSCs: cancer stem cells
circRNA: circular RNAs
DINO: damage-induced non-coding
DDR: DNA damage response
DNMTs: DNA methyltransferases
DSBs: double-strand breaks
EMT-TF: EMT transcription factors
EZH2: enhancer of zeste homologue 2
EGFR: epidermal growth factor receptor
EMT: epithelial-to-mesenchymal transition
HPVs: human papillomaviruses
HR: high-risk
IGF2BP1: insulin-like growth factor 2 mRNA-binding protein 1
JAK/STAT: Janus Kinase/Signal transducer and activator of transcription
MAPK: Mitogen-activated protein kinase
MBD: methyl-binding domain
METTL3: methyltransferase-like 3
MMEJ: microhomology-mediated end-joining
NHEJ: nonhomologous end-joining
NOTCH: Neurogenic locus notch homolog protein
NuRD: nucleosome remodelling and deacetylation
OCT4: Octamer-binding transcription factor 4
SMAD: Mothers against decapentaplegic homolog
TET1: ten-eleven translocation enzymes 1
TMPOP2: thymopoietin pseudogene 2
TAZ: Transcription Adaptor putative Zinc finger
TGF: Transforming growth factor
TGFβ: Transforming growth factorβ
TWIST: Twist-related protein 1
YAP1: Yes-associated protein 1
ZEB1: Zinc finger E-box-binding homeobox 1
m6A: N6-methyladenosine
ncRNA: non-coding RNAs
